# Characteristics of equestrian accidents and injuries leading to permanent medical impairment

**DOI:** 10.1186/s13102-024-00973-8

**Published:** 2024-09-04

**Authors:** Helena Stigson, Maria Klingegård

**Affiliations:** 1 Folksam Research, Folksam Insurance Group, Stockholm, Sweden; 2 Division of Insurance Medicine, Department of Clinical Neuroscience, Karolinska Institutet, Stockholm, Sweden

**Keywords:** Equestrian, Injury, Injury incidence, Prevention

## Abstract

**Background:**

Equestrian sports, also referred to as equestrianism, is practiced all over the world and a popular leisure activity in Sweden. Equestrianism is the country’s second-largest youth sport, and previous studies indicate that accidents are common in equestrianism. However, few previous studies have examined acute equestrian injuries leading to permanent medical impairment (PMI).

**Methods:**

By using nationwide insurance data comprising all injured equestrians registered in the Swedish Equestrian Federation, the aim was to analyse characteristics of equestrian accidents and particularly injuries leading to PMI, both in general and by age, gender, incident type, injury type and injured body region. Injury incidence and injuries leading to PMI were examined. All equestrians injured during 2017–2021 were included (*n* = 6,876), however, only injuries occurring in 2017 and 2018 were used to analyse the risk and the injury distribution of injuries leading to PMI. Injury incidence was calculated separately for males and females, as well as for different age groups, by dividing the number of insurance claims by the number of members multiplied by 1,000. Risk Ratio (RR) was calculated between gender. Pearson’s chi-squared test was used to analyse differences in distribution for categorical variables.

**Results:**

The majority of injuries affected females, correlating to approximately three times higher risk, compared to males. The age group 21–40 years were associated with the highest injury risk (14.26/1,000 registered riders). In total 12% of all injuries led to PMI. Injuries to the upper and lower extremities, along with fractures, had a higher risk of resulting in a PMI. The risk of injury leading to PMI was higher among riders over 20 years of age.

**Conclusions:**

The fact that females face nearly three times the injury risk of males, and riders aged 21–40 had the highest injury risk while younger riders (Luke KL, McAdie T, Smith BP, Warren-Smith AK. New insights into ridden horse behaviour, horse welfare and horse-related safety. Appl Anim Behav Sci. 2022;246:105539.); (Havlik HS. Equestrian sport-related injuries: a review of current literature. Curr Sports Med Rep. 2010;9(5):299–302.); (Samuels K, Bettis A, Davenport DL, Bernard AC. Occupational vs. non-occupational equestrians: Differences in demographics and injury patterns. Injury. 2022;53(1):171–5.); (Gharooni A-A, Anwar F, Ramdeep R, Mee H. Severe equestrian injuries: A seven-year review of admissions to a UK major trauma centre. Trauma. 2023;25(1):41–7.); (Hasler RM, Gyssler L, Benneker L, Martinolli L, Schotzau A, Zimmermann H, et al. Protective and risk factors in amateur equestrians and description of injury patterns: A retrospective data analysis and a case - control survey. J Trauma Manag Outcomes. 2011;5:4.); (Meredith L, Brolin K, Ekman R, Thomson R. Analyses of injuries to equestrians in a Swedish district over a 16-year period. Translational Sports Med. 2019;2:270–8.) had a lower risk, indicates that preventive efforts should target both older and female riders. Injuries predominantly resulting in PMI involve upper and lower extremities, however, to prevent the most serious injuries significantly affecting a rider’s daily life, measures preventing head and spinal cord neck injuries, must be implemented. Head injures remain the most frequent, serious and most significant group of injuries to prevent and mitigate, within equestrian sports.

## Background

Equestrianism, a popular activity in Sweden since the mid-twentieth century [1], offers numerous physical [2] and psychological benefits [3]. It is enjoyed both recreationally and therapeutically, providing muscular exercise and improved balance for a healthier lifestyle. However, globally, horse riding is emerging as a significant public health concern, with statistics showing an alarming frequency of acute injuries and even fatalities linked to equestrian accidents [4–6]. Unlike many other sports, riding is a high risk sport due to the elevated position of riders on powerful horses, horse speed, and the unpredictability of horse behaviour [7], making it different to many other sports. Risk factors may be influenced by the higher number of female riders, especially at the recreational and amateur levels [8–11].

Previous studies on Swedish equestrian injuries show that accidents affect all ages [6, 12–14], although the highest risk of injury is found among riders aged 21–40 years (8.83/1000 registered riders) [13]. On average, three fatal accidents occurred annually between the years 1997–2014 [14]. Several studies have shown that the rider is usually injured due to falling off the horse [6, 10, 13, 15]. Other common causes of injury include the rider being bitten, kicked or trampled on. An overview by Meredith et al. [15] shows that the type and location of injury depend on the activity performed (e.g. riding and handling a horse). In addition, the injury pattern changes with the rider’s age [6, 13, 14, 16]. For example, Franzén et al. [6] demonstrated that as age increases, the risk of upper extremity injuries decreases, while the risk of vertebral fractures and thoracic injuries rises. In general the head is the most frequently injured region [17] and equestrian sports have been identified as one of the predominant contributors to sports-related traumatic brain injury [18]. Furthermore, almost half of equestrian riders (44%) were subject to concussion during their career [19]. Equestrians are exposed to both acute injuries and overuse injuries. Meyer et al. [20] found that overuse injuries among German show jumpers are relatively infrequent and primarily affect the upper extremities. Additionally, a study of Swedish eventing athletes revealed that acute injuries are more common than overuse injuries [21].

Few studies have had the opportunity to study if long-term consequences arise from equestrian injuries. The existing studies primarily focus on serious injuries that have required hospital care often treated at a trauma centre. Hence, the percentage of injuries resulting in long-term consequences, is as high as up to 50% of all injuries (see for example [22–25]). The Swedish insurance company, Folksam Insurance Group (Folksam), has been the appointed insurer for all members of the Swedish Equestrian Federation since 2017, providing the possibility of verifying and following up on injuries leading to long-term consequences, denoted as permanent medical impairment (PMI). Since all members are included, the data set is suitable for evaluating incidence rates. Studies into equestrian injuries leading to PMI a currently lacking. Grading of medical impairment degree [19] principles, established in the early 20th century in consensus between physicians, are applied by Swedish insurance companies. PMI is assigned a percentage between 1 and 99, regardless of the individual’s occupation or hobbies. Examples of impairment degrees include: 7% for an unstable ankle joint, 5–20% for a shoulder with maximal flexion of 0-120°, 37% for loss of one hand, 68% for total blindness, and 99% for severe dementia. Previous studies have shown that PMI can be used to predict which injuries should be prioritised to prevent lifelong complications (see for example [26, 27]). Hence, it is crucial to study the long-term consequences of rider injuries, being essential for riders’ overall well-being, the sustainability of sports careers, and the development of effective preventive actions.

The aim of this study was to analyse characteristics of equestrian accidents and particularly injuries leading to PMI, both in general and by age, gender, incident type, injury type and injured body region.

## Materials and methods

Folksam offers comprehensive insurance coverage to all members of the Swedish Equestrian Federation, covering everyone from beginners to competitive athletes. The insurance covers every member during training, Swedish Equestrian Federation activities, as well as competitive riding. This five-year retrospective study includes all horse-related injuries among all members of the Swedish Equestrian Federation reported to Folksam between 1 January 2017 and 31 December 2021 (*n* = 6,876). From 2017 to 2021 the number of registered members has increased from 138,511 to 143,516. Both exposure on an individual level and injury data were used.

All eligible injuries, defined as an acute injury reported to the sport injury database of Folksam, occurring while participating in an activity with, or related, to horses. This definition comprises both horse riding and horse care activities, in conjunction with either training or competition. An acute injury is defined as an injury resulting from a specific identifiable event. Overuse injuries, resulting from repetitive microtrauma to tissues over time without sufficient recovery are not covered by the insurance, and therefore not included in the study. Injuries were reported by the rider, or a parent of minor riders, via telephone or online. In the present study, all riders undergoing a medical examination within one week of the accident, were included. Consequently, all injuries, regardless of severity or the healthcare they received, have been included. While the majority of injuries were diagnosed by physicians, some were diagnosed by other experts, such as physiotherapists. The injuries were categorised into different categories based on type of injury and body location. The injured body region was categorised into five groups: head and neck (including face), torso, upper extremities, lower extremities and unknown. The type of injury was based on the classification made by claims adjusters including the following seven groups: fracture, dislocation/rupture, sprain/strain, laceration/abrasion/contusion, concussion, and “multiple injury types”. The category “multiple injuries types” includes riders who have sustained multiple injuries simultaneously.

An acute injury can lead to a PMI, resulting in lifelong complications [28]. A total of 357 personal injuries resulted in PMI in the years 2017 to 2021. However, as determining whether an injury will result in permanent impairment takes at least two years, in some cases significantly longer, only injuries occurring in 2017 and 2018 were used to analyse the risk and injury distribution of injuries leading to PMI. The incidence of PMI was calculated as a proportion of all registered injuries during these two years. A preliminary degree may be assigned before establishing PMI due to the challenge of accurately predicting the long-term consequences of an injury, especially among young people or in cases of specific diagnosis such as brain injuries with long rehabilitation Therefore, both preliminary and permanent degrees are included in this study.

Data comprised information on age (0–6, 7–12, 13–20, 21–40, 41 years or older, predefined by the Swedish Equestrian Federation) gender, date of injury, nature of injury and injured body region. Additionally, claimants were required to provide a description of the injury event, which was then categorised into type of activity (horse riding, horse handling, mounting or dismounting or other) and incident type (caught in equipment, falls, kicks, trampled or “other” (including for example being bitten, crushed, and knocked by horse). The study has been approved by the Swedish Ethical Review Authority (Dnr 2018/711 − 31/5). Participant consent is governed by the insurance agreement, which includes a provision requiring their consent to contribute to research. As such, individual consent from each injured participant was not required.

### Statistical analysis

Descriptive statistics, including frequencies and percentages, were conducted on all available variables using IBM SPSS Statistics version 28. The injury incidence was calculated separately for males and females, as well as for different age groups, by dividing the number of insurance claims by the total number of members and multiplying the result by 1,000. This is expressed as “the number of injuries per 1,000 members”. The Risk Ratio (RR) was calculated to compare the risk between gender, obtained by dividing the injury incidence in females by that in males [29]. Pearson’s chi-squared test was used to analyse differences in distributions for categorical variables. All analyses were two-sided, with a significance level set at *p* < 0.05.

## Results

**Fig. 1:**
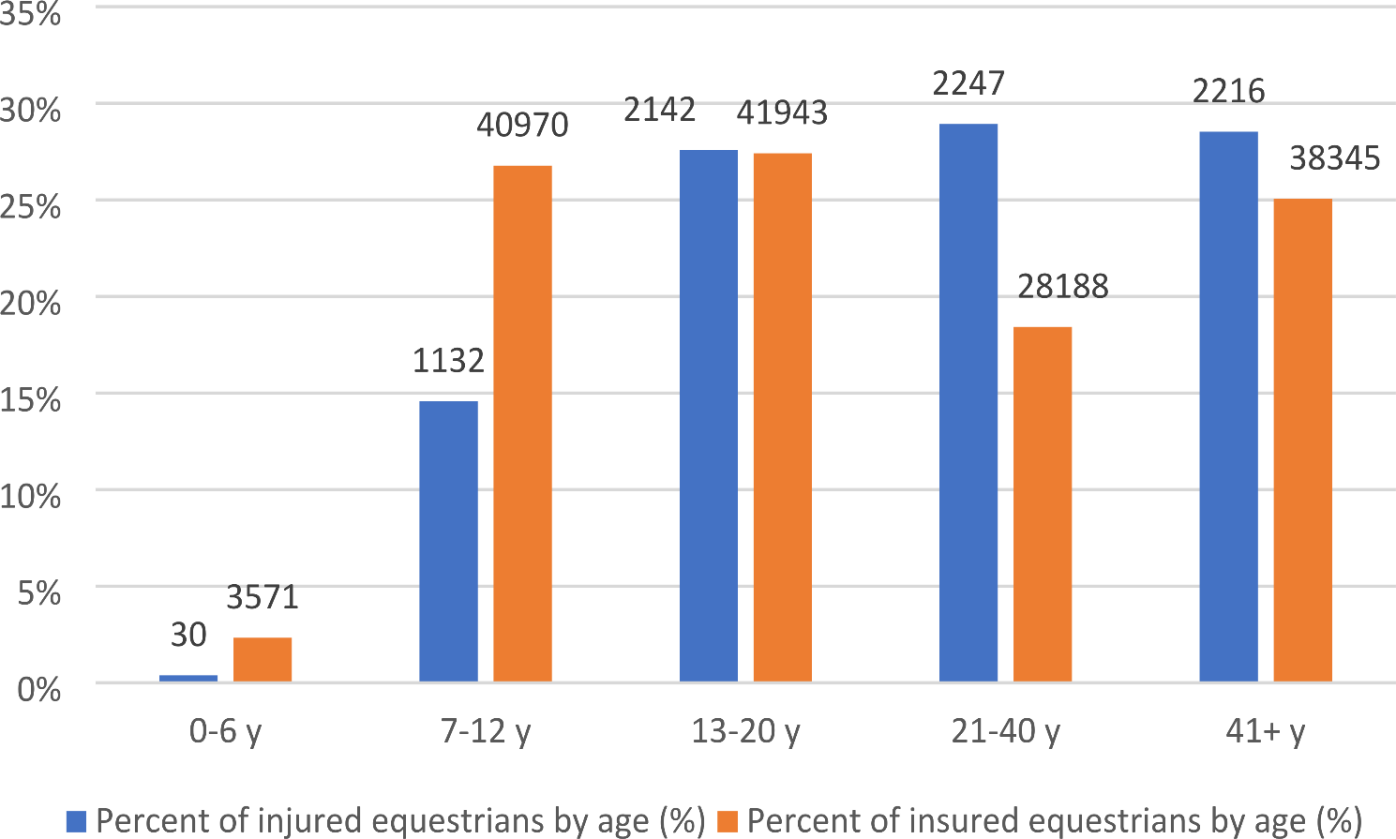
Distribution of injuries and total number of insured riders per age category (average number of members in the years 2017 to 2021)

During the period 2017 and 2021, 6,876 riders made insurance claims to Folksam following an acute traumatic injury while participating in horse riding or horse care activities, Table 1. Among the 6,876 equestrians involved in accidents, a total of 7,768 injuries were reported, resulting in an average of 1.1 injuries per individual. Within the study period, a total of three riders were fatally injured. In total, female riders predominated, with 97% of the injured equestrians being female. The average age of injured riders was 29 years, ranging from 3 to 80 years. The average age of men was higher compared to women. The average age for women was 29 years (ranging from 6 to 75 years), whereas for men it was 38 years (ranging from 3 to 80 years). The percentage of riders injured in the different age-groups compared with all registered members can be seen in Fig. 1. The proportion of injuries was highest among older age groups.

**Table 1:** Characteristics of equestrian injury accidents in 2017–2021,

Variable	All	Female	Male
Number of Injured Riders	6876	6658	218
Number of Injuries	7779	7539	240
Age, mean (range)	29 (3–80)	29 (6–75)	38 (3–80)
Type of Accident			
*Falls*	84%	80%	84%
*Kicks*	4%	7%	4%
*Entanglement in Equipment*	3%	2%	3%
*Trampled*	2%	3%	2%
*Other*	7%	8%	7%
Type of Activity			
*Riding*	87%	84%	87%
*Horse-handling*	9%	10%	9%
*Mounting or Dismounting.*	2%	3%	2%
*Other*	2%	3%	2%

### Incident type and type of activity

The predominant incident type was falls from horseback, making up 84% of incidents, followed by kicks at 4%, entanglement in equipment at 3%, and being trampled by a horse at 2%, Table 1. Among riders with multiple injuries, a slightly higher proportion of featured incidents falling off the horseback while riding (88%). Falls were the primary cause of injuries during riding, whereas kicks usually occurred during horse-handling and mounting/dismounting activities. In total, 87% of the injuries occurred while riding, 9% during horse-handling and 2% while mounting/dismounting, Table 1.

### Body location

Aggregated data on body region shows that the most injured body region was the “head and neck” (44%) followed by “upper extremity” (28%), Table 2.

**Table 2:** Distribution of injuries and injuries leading to permanent medical impairment (PMI) by body region

Body Region	Number and proportion of Injuries (based on all cases)		Number of Injuries Resulting in PMI* (based on injuries that occurred 2017–2018)
**Head and Neck injuries**	2,996		34
Head/brain	605	20%	7
Concussion	1,215	41%	8
Face	254	8%	8
Eye	19	1%	-
Tooth	121	4%	-
Cervical Spine	157	5%	5
Riders with multiple injuries including head/ neck injuries	577	19%	6
Unknown	48	2%	-
**Upper Extremity**	1,950		133
Shoulder	758	39%	29
Upper Arm	160	8%	15
Elbow	103	5%	10
Forearm	100	5%	10
Wrist	266	14%	23
Hand/Finger	487	25%	45
Riders with multiple injuries including upper limb injuries	34	2%	1
Unknown	42	2%	-
**Torso**	1,139		50
Sternum/Ribs/Upper Back	651	57%	26
Abdomen incl. Organs	125	11%	1
Lower Back/Pelvis/Sacrum	212	19%	16
Spine, undefined	55	5%	3
Riders with multiple injuries including torso injuries	69	6%	4
Unknown	27	2%	-
**Lower extremity**	1,592		97
Hip/Thigh	712	45%	19
Knee	259	16%	20
Lower Leg	160	10%	19
Ankle	202	13%	24
Foot/Toe	211	13%	14
Riders with multiple injuries including lower extremity injuries	25	2%	1
Unknown	23	1%	-
**Unknown Body Region/Other Type of Injury**	91		1
Total Number of Injuries	7,768		314
Total Number of Equestrians	6,876		297

*Permanent Medical Impairment

### Injury type

**Fig. 2:**
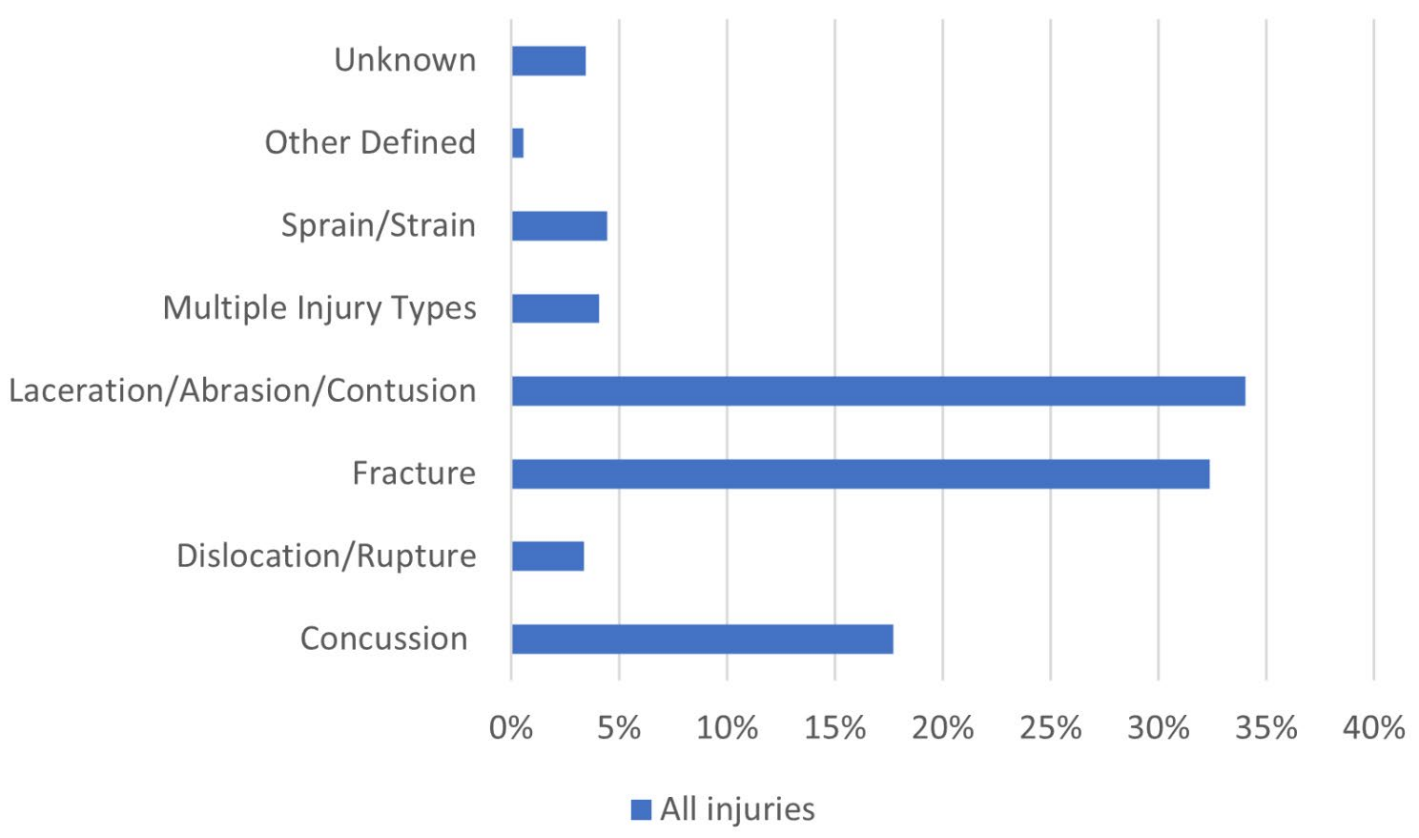
Distribution of the different types of injuries based on all injuries 2017–2021

The most frequently seen injury types were soft tissue injuries such as laceration, abrasion, and contusion (34%) and fractures (32%), Fig. 2. Concussion was also commonly occurring, accounting for 18% of all injuries. Fractures were higher among riders older than 20 years of age (*p* < 0.001). There was a higher proportion of concussion among female riders (*p* = 0.038), however, gender did not make a difference with regard to fractures.

### Injuries leading to PMI

In total, 12% of all injured riders sustained a PMI during the years 2017 and 2018. Of these, 42% involved an injury to the upper extremities, Table 2. Fingers, followed by a shoulder, were the most common parts of the upper extremities affected. The highest risks of sustaining a PMI for an injury was found for the upper and lower extremities (19% and 18% respectively), Table 3. However, head and neck injuries often resulted in a higher average degree of PMI than other injuries. The majority of riders who had sustained PMI of 10% or more, had suffered a brain injury or damage to the spine.

**Table 3:** Number of injuries, injuries leading to PMI, risk of PMI and average degree of PMI by body region

Injured Body Region	Total Number of Injuries (during 2017–2018)	Number of injuries leading to PMI* (during 2017–2018)	Risk of PMI* (%)	Average degree of PMI* (min-max)
Head and Neck	961	34	4	12(1–90)
Upper Extremity	717	133	19	3(1–9)
Torso	471	50	11	4(1–14)
Lower Extremity	551	97	18	3(1–14)
Spine**	242	28	12	7(1–90)
Other injuries	16	1	6	7(-)
Total Number of Injuries	2,958	314	11	4(1–90)
Total Number of Riders	2,450	297	12	4(1–90)

*Permanent Medical Impairment

**Included as a subset in Head and Neck, Torso and Lower Extremity

**Fig. 3:**
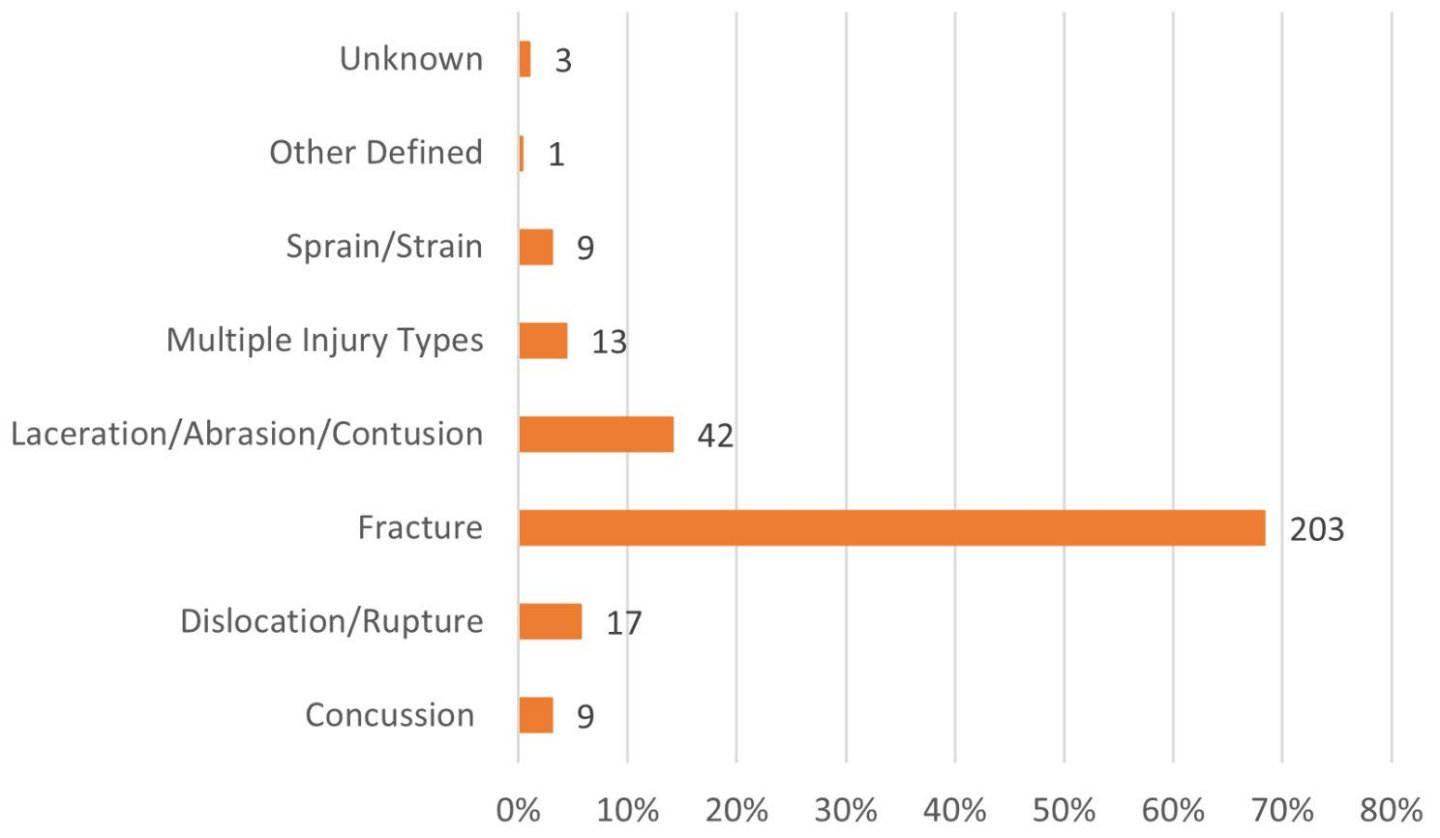
Distribution of different types of injuries showing injuries leading to PMI

Fracture was the most common type of injury leading to PMI, Fig. 3. The risk of a fracture resulting in PMI was higher compared to other injuries (*p* < 0.001). Furthermore, riders with multiple injuries had a higher risk of either of the injuries resulting in PMI, than riders with only one injury (*p* = 0.01). There was no difference between genders if an injury resulted in PMI. However, a higher risk of injury resulting in PMI was higher among riders older than 20 years of age (*p* < 0.001). The average age of a rider with an injury resulting in PMI was 37 years.

The majority of injuries resulting in PMI occurred during riding activities (74%), primarily resulting from falls off a horse (93%). About 18% of PMI cases stemmed from horse handling, with kicks or entanglement in equipment being the primary causes. Irrespective of the activity, falls constituted the most common cause of injury (70%), followed by being kicked by the horse (7%) and getting caught in equipment (7%).

### Injury incidence

The overall injury incidence rate was 8.98 injuries per 1,000 registered riders per year, Table 4. Females were associated with an almost three times higher risk of injury than males (RR = 2.71) in general, at an injury incidence of 9.47 compared with 3.49 for males and more than three times higher risk of an injury resulting in PMI (RR = 3.47, 2.08 for females and 0.6 for males). Riders aged 21–40 years were associated with the highest injury incidence (14.25/1,000 registered riders), while 0-6-year-olds had the lowest (1.51/1,000 registered riders). Riders aged 13–20 years or 21–40 years were associated with the highest incidence of concussions (2.27–2.33/1,000 registered riders), although the proportion of concussions were highest among riders younger than 21 years of age (*p* < 0.001).

**Table 4:** Injury incidence, concussion incidence and PMI incidence for different age groups and genders

Category	Injury Incidence	Concussion Incidence	PMI* Incidence
0–6y	1.51	0.28	
7–12y	4.90	1.02	
13–20y	8.94	2.27	
21–40y	14.25	2.33	
41 + y	10.18	1.02	
Female	9.47	1.69	2.08
Male	3.47	0.43	0.60
Total	8.98	1.59	1.95

*Permanent Medical Impairment

## Discussion

In this retrospective cohort study evaluating injured members of the Swedish Equestrian Federation spanning all age groups, it was found that around 1,400 riders annually, had reported equestrian injuries via their insurance. This corresponds to approximately 26 injured riders per week. Notably, injures affect riders across all ages, with the average age of injury being 29 years. Although, it is often highlighted that younger riders sustain injuries [6, 12, 30], which holds true in terms of raw injury numbers, when injured riders are put in relation to the proportion of practitioners in each age group, the results from this study uncover a higher risk of injury among older riders.

In previous studies such as Meredith et al. [12] and Franzén et al. [6], who studied admissions to healthcare facilities in one region of Sweden and one hospital trauma registry, they showed that most injured people were aged 10 to 21 years. Furthermore, another Swedish study estimated that approximately 9,000 of riders, injured annually in equestrian activities, required visiting an Accident & Emergency department, and that the largest proportion of injured riders were found in the 11–17 age group [31]. The average age of injured riders was 24 years in Franzén et al. [6] and 27 years in Meredith et al. [14], with the highest number of injuries in the age group 0–18 years. It is important to consider that these studies are based on all individuals who sought medical care after a horse riding and/or handling accident and are not in relation to the number of individual’s practicing these activities on a regular basis. Furthermore, it remains unclear how many of these riders were injured due to activities within the Swedish Equestrian Federation. In the present study, the results are based on Folksam injury data, which only comprises members of the Swedish Equestrian Federation and cases the data was set in proportion to injuries in relation to the number of members.

However, there are some international studies showing that both the number of injuries and the risk of being injured differ for different riding ages [13, 16, 30, 32]. Similar to the present study, Smartt and Chalmers [32] found comparable average ages and noted that females tended to be younger and more frequently injured than males. However, the incidence rates were based on general participation in equestrian activity and not membership of an equestrian federation. This difference presents a challenge when directly comparing these findings to the data in the present study. Acton et al. [33] demonstrate parallels with the present study in that most horse-related injuries were diagnosed as a fracture or soft tissue injury. Furthermore, similarities were also found when studying female riders, who were more likely to have sustained a head and neck injury as well as being diagnosed with a concussion, compared to male riders. Similarly, younger riders were more likely to sustained a head and neck injury or be diagnosed with concussion, than older riders. In the present study older riders had a higher risk of both injuries and injuries leading to PMI. This aligns with findings from a study using German data [17], emphasising the importance of considering older individuals as a target group in efforts aiming at preventing serious injuries. A literature study by Meredith et al. [15], however, shows few studies indicating that older, rather than younger riders, have a higher injury rate. This statistic is mainly due to more young riders being included in previous studies. Acton et al. [33] highlight the need for more studies including older riders. Therefore, it is recommended that further studies explore the increased injury risk among older riders and investigating other injury characteristics in further detail, which could provide deeper insight into effective preventive strategies.

Regarding equestrian injuries leading to long-term consequences, Ball et al. [25] showed that a high number of orthopaedic related injuries resulted in residual functional deficits, supporting the finding of the present study showing that fractures more often resulted in PMI compared to other injuries. In total 12% of all the injured riders during the years 2017 and 2018 sustained a PMI. While this figure might appear low in contrast to a previous study [25], it is important to note that the present study includes all injuries, irrespective of healthcare and injury severity. PMI was higher among riders older than 20 years of age, for several reasons. A medical impairment is considered permanent when no further improvement in physical and/or mental function is expected with additional treatment. Medical impairment injury is monitored until the permanent degree of medical impairment can be determined, which in some cases take several years and cannot always be established until adulthood. This might explain why young riders had a lower risk of an injury resulting in PMI. However, previous studies have shown that in general, the risk is higher for older individuals [34, 35]. Compared to studies involving individuals from other sports federations, such as the Swedish Cycling Federation, the risk of an injury sustained in competitive cycling resulting in PMI was lower than that observed among equestrians (9% compared to 12%) [36]. As a rider, you sit approximately 3 m above the ground, on an animal that can easily weigh 500 kg or more. High speed combined with falling from a great height is a major risk factor for severe injuries, not found in many other sports and activities.

In similarity with several studies, the most common incident was falls from horseback [13, 16, 30, 32, 33]. Falls from horses also constitute the majority of accidents involving injuries leading to PMI. However, when studying injuries leading to PMI, the proportion of accidents occurring while handling a horse, with kicks or entanglement in equipment being primary causes, becomes more significant (18% compared to 9% of all injuries). Hence, it is crucial to also consider that handling a horse presents a notable risk of injury. This aspect has been highlighted by Krüger et al. [17] and should be included when devising injury prevention strategies. For instance, adopting safety measures like wearing helmets and body protectors during horse-handling could effectively minimise the risk of injuries.

One of the main strengths of this study is the use of high quality insurance register data, with exposure data for the total Swedish Equestrian Federation population coverage and follow-up over several years. As mentioned, at the earliest, the PMI is assessed by the insurance company two years following an injury, thus PMI has only been based on data for the years 2017 and 2018. If the policyholder has an agreement with more than one insurance company, all companies will compensate for the PMI but not for the acute injury. This might exaggerate the risk of injuries leading to PMI.

The protective effects of equestrian helmets for head and facial injuries have been confirmed [11]. In the present study, head and neck injuries accounted for 44% of all injuries and it could therefore be argued that current helmet designs are not efficient enough. However, one limitation with the present study includes not being able to investigate all accidents in which riders were wearing a helmet. The insurance data only allowed tracking of cases where compensation was claimed due to damaged helmets. Nonetheless, several studies have stated that the protective effect of helmets must be improved and should be designed specifically for the softer ground and impact surfaces typically seen in falls from horsebacks [37–39]. During Swedish Equestrian Federation events, riders are required to wear a helmet while riding, and the recommendation of wearing a helmet while on the ground, is becoming more common. This recommendation should be further encouraged and enforced.

In the present study, only acute injuries were included. To gain a comprehensive understanding of all injuries among equestrians, overuse injuries should also be studied [20, 21]. Furthermore, it has not been feasible to examine acute injuries across the various disciplines of equestrian sport, which would benefit from being investigated in future studies.

## Conclusions

This nationwide register study shows that annually, approximately 1,400 out of 155,000 members within the Swedish Equestrian Federation, made a claim following an acute traumatic injury while participating in a horse-related activity. Therefore, the incidence of being injured is nine injured riders per 1,000 insured. The majority of injuries affected females, correlating with an almost three times higher risk compared to males.

Younger riders are often highlighted as the most injury-prone group. However, this study highlights that riders aged 21–40 years were associated with the highest risk of injury, whereas young riders (7–12 years old) had a comparatively lower risk. Furthermore, the injury distribution differs by age group and injuries resulting in PMI is higher among older riders. Hence, preventive efforts should target older riders as well.

This study further confirms that falls from horses are the single most significant cause of equestrian accidents, however, handling a horse also presents a notable risk of injury. Approximately, 12% of injuries led to PMI. Injuries to the upper and lower extremities, along with fractures, had a higher risk of resulting in a PMI. However, to prevent the most serious injuries significantly affecting a rider’s daily life, measures to prevent head and spinal cord neck injuries should be implemented. Therefore, head injures remain the most frequent, serious and the most important injuries to prevent and mitigate within equestrian sports.

## Data Availability

The data that support the findings of this study are available from the corresponding author upon reasonable request.
